# Supplementary material for: A Simple Synthesis Route for Selectively Methylated β-Cyclodextrin Using a Copper Complex Sandwich Protecting Strategy
Source: Molecules. 2021 Sep 18;26(18):5669. doi: 10.3390/molecules26185669 (PMC8466452; doi:10.3390/molecules26185669)
Supplement: Supplementary file 1 [file molecules-26-05669-s001.zip › molecules-1369115-supplementary.pdf]

Supporting information for:

A Simple Synthesis Route for Selectively Methylated *β*-cyclodextrin Using a Copper Complex Sandwich Protecting Strategy

Stefan Bucur[1,*], Marius Niculaua[2], Catalina Ionica Ciobanu[3], Neculai Catalin Lungu[1], Ionel Mangalagiu[1,3,*]

[1] Faculty of Chemistry, Alexandru Ioan Cuza University of Iasi, 11 Carol 1st Bvd, 700506 Iasi, Romania;

[2] Research Center of Oenology, Romanian Academy—Iași Division, 9th M. Sadoveanu Alley, 700505 Iași, Romania

[3] Institute of Interdisciplinary Research- CERNESIM Centre, Alexandru Ioan Cuza University of Iasi, 11 Carol I, Iasi, 700506, Romania

**E-mail address: bucurm.stefan@gmail.com; stefan.bucur@chem.uaic.ro (S.B.) ionelm@uaic.ro (I.M.)*

S1. NMR and MALDI-TOF Spectra

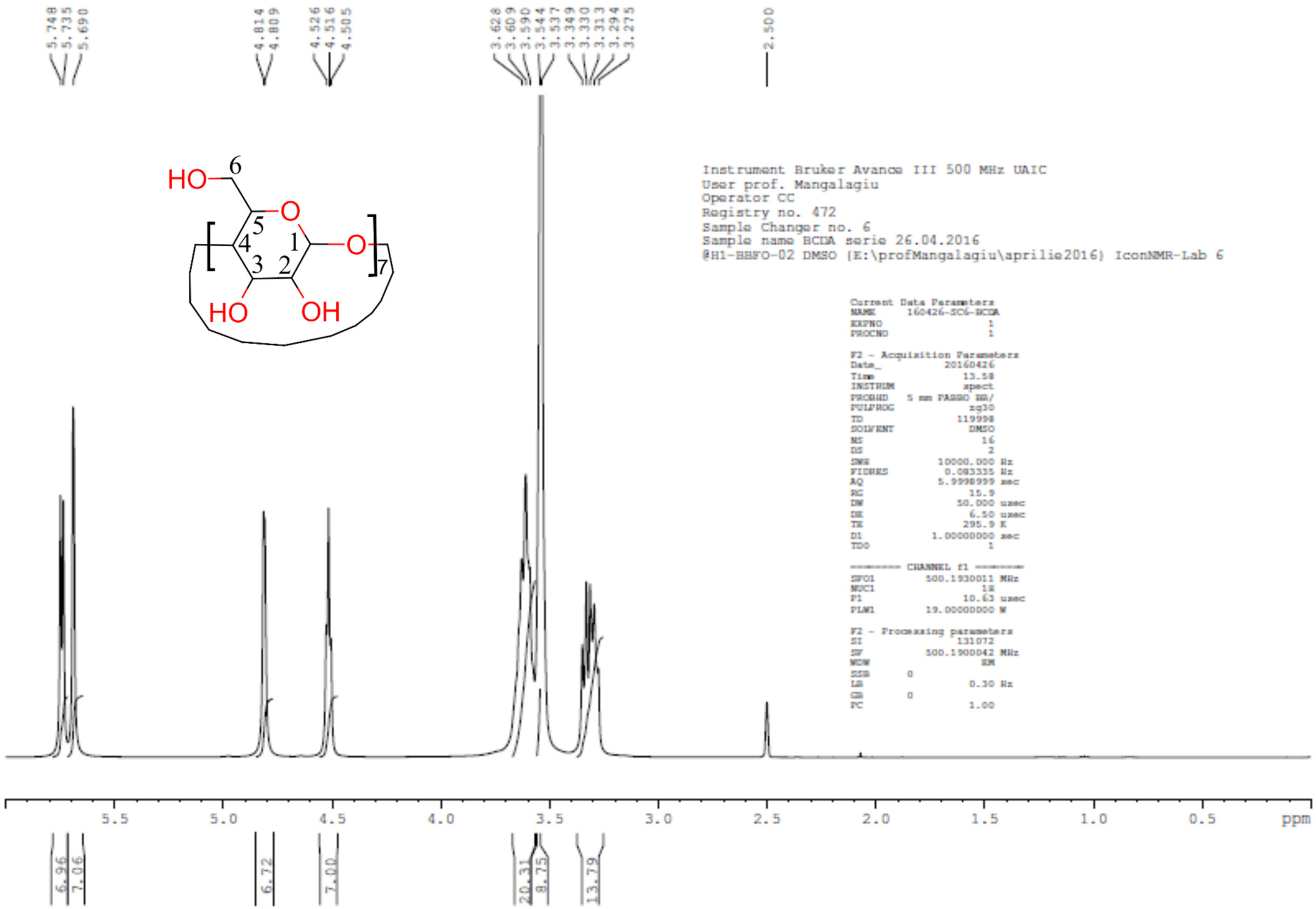

Figure S1. ^{1}H-NMR spectrum of β-CD

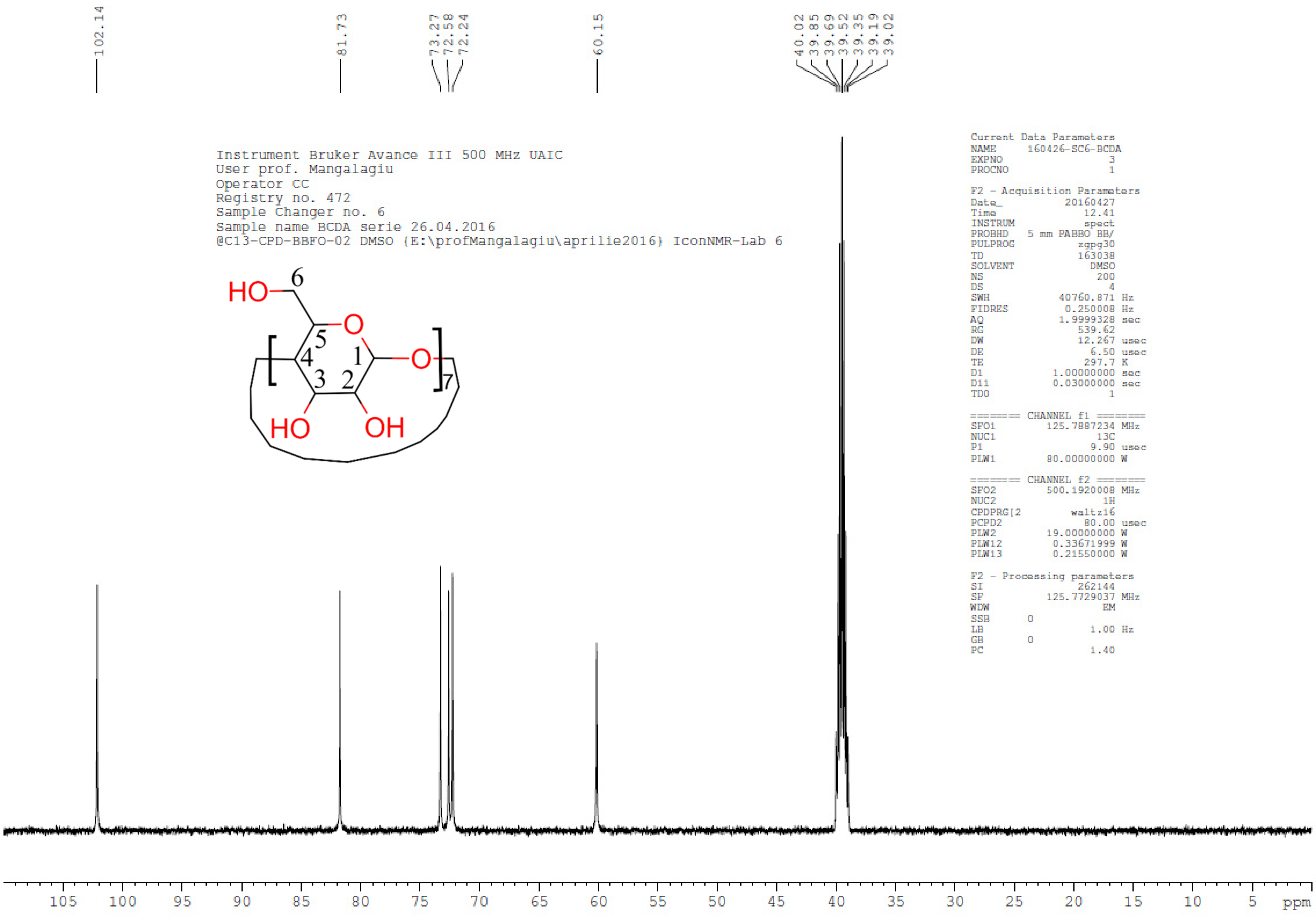

Figure S2. ^{13}C-NMR spectrum of β-CD

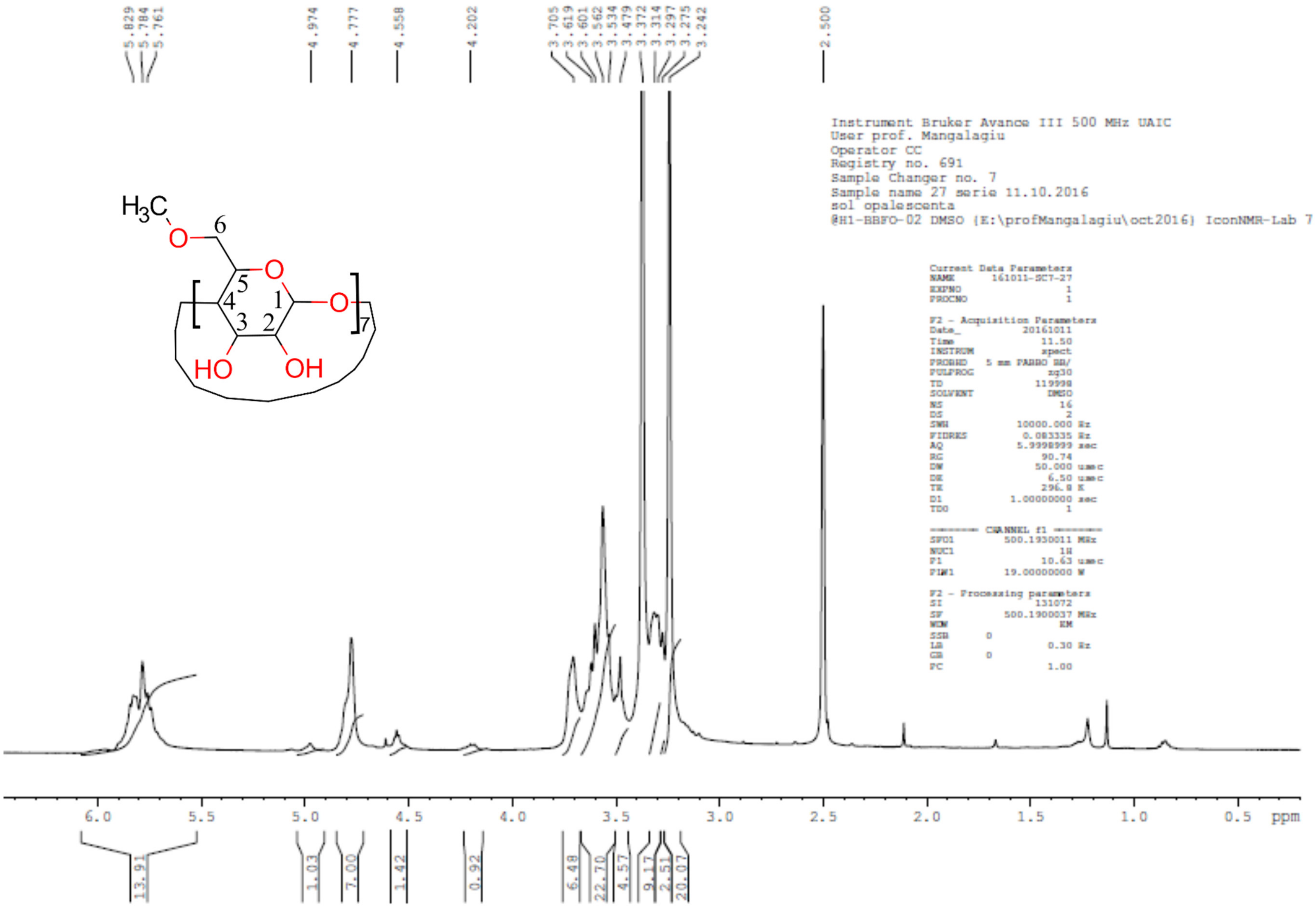

Figure S3. ^{1}H-NMR spectrum of MβCD

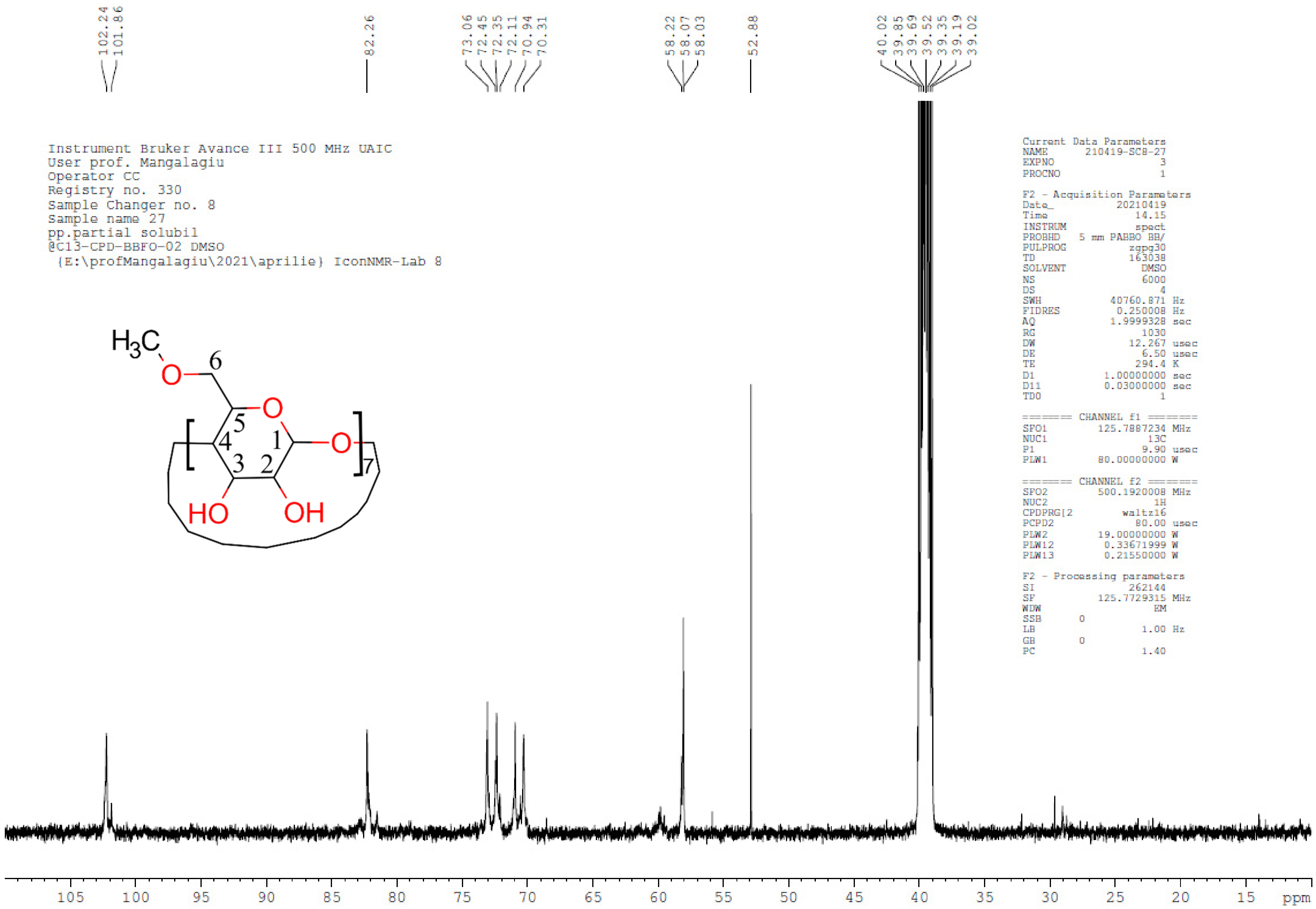

Figure S4. ^{13}C-NMR spectrum of MβCD

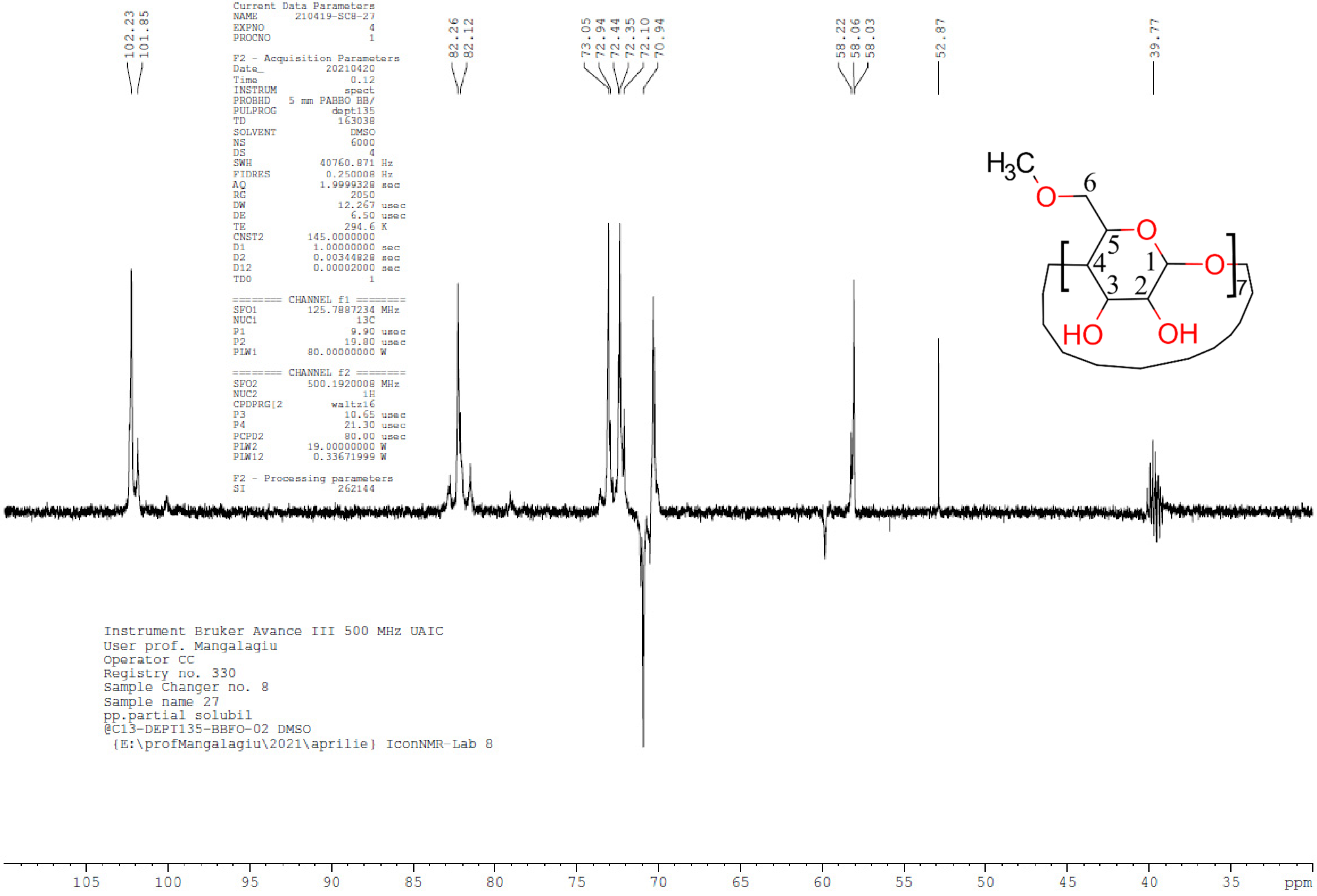

Figure S5. DEPT-135 ^{13}C-NMR spectrum MβCD

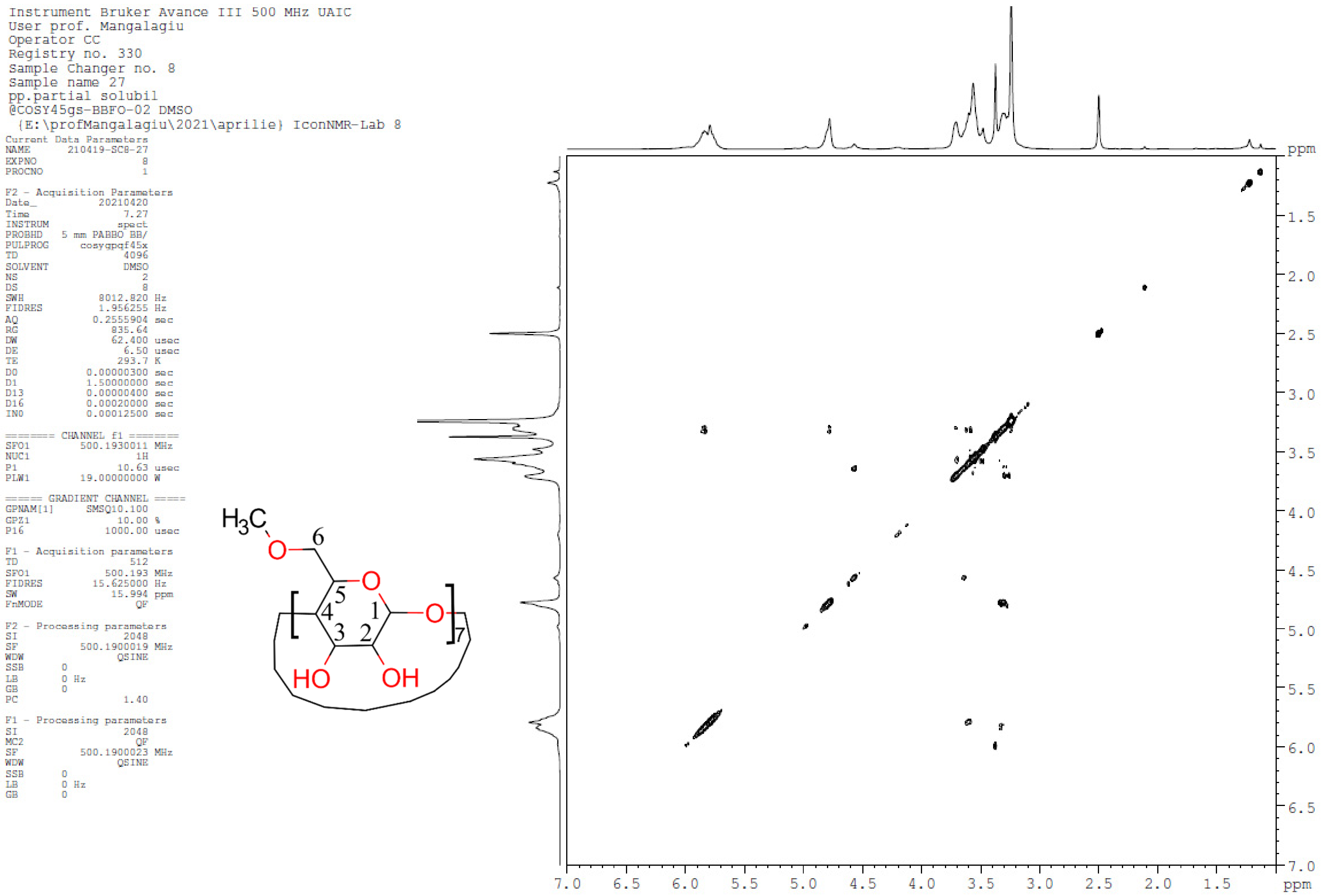

Figure S6. COSY NMR spectrum MβCD

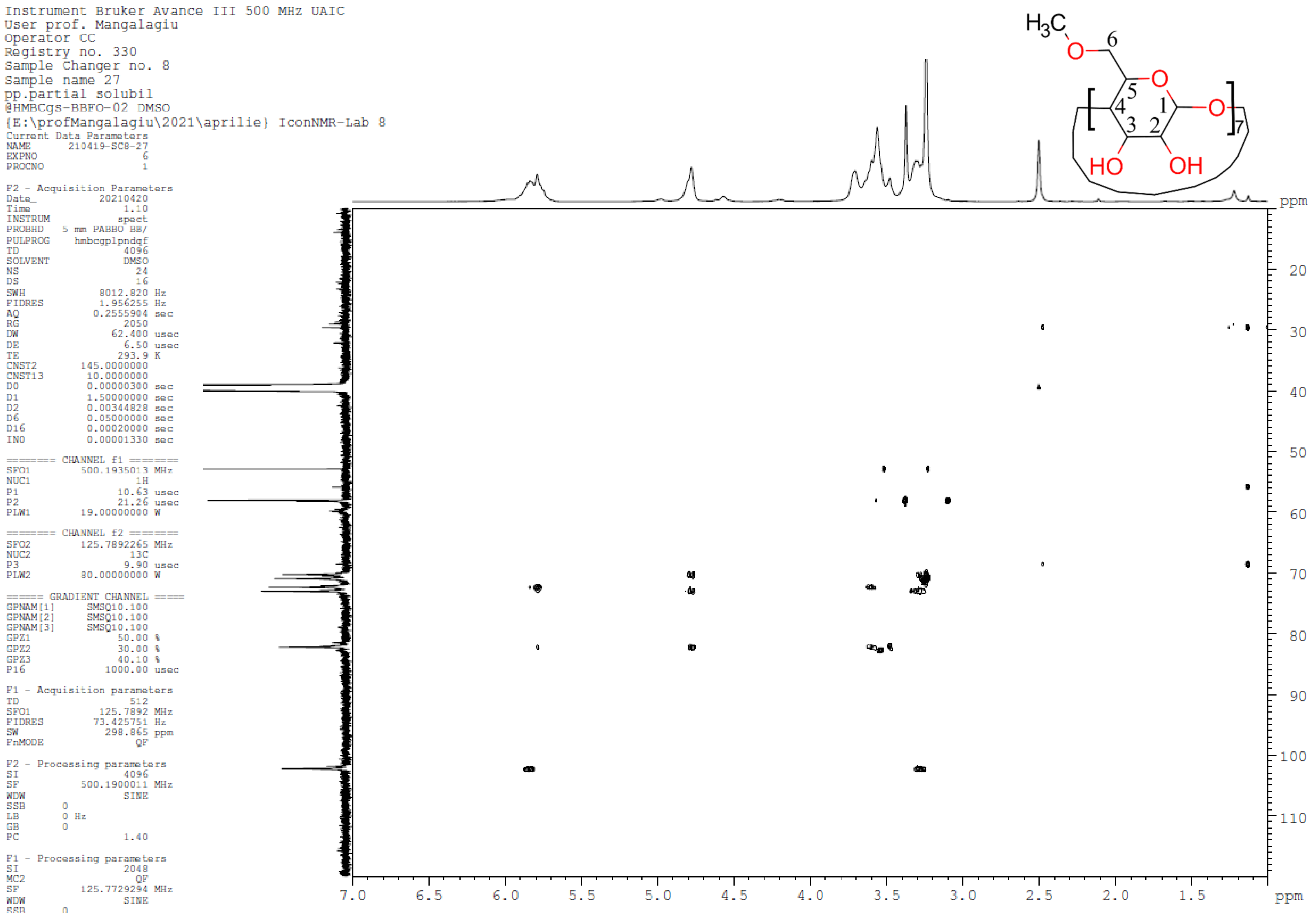

Figure S7. HMBC NMR spectrum MβCD

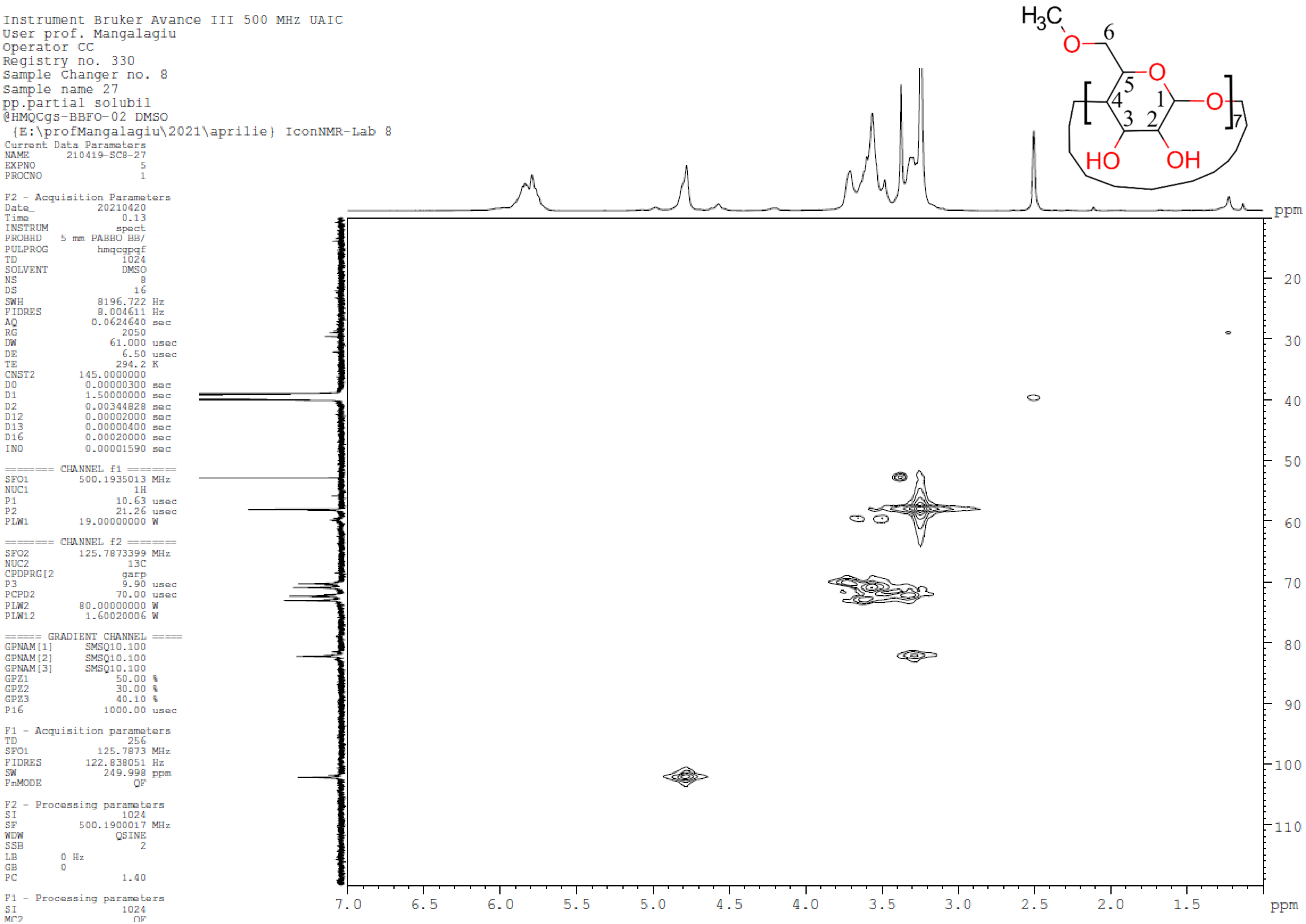

Figure S8. HMQC NMR spectrum MβCD

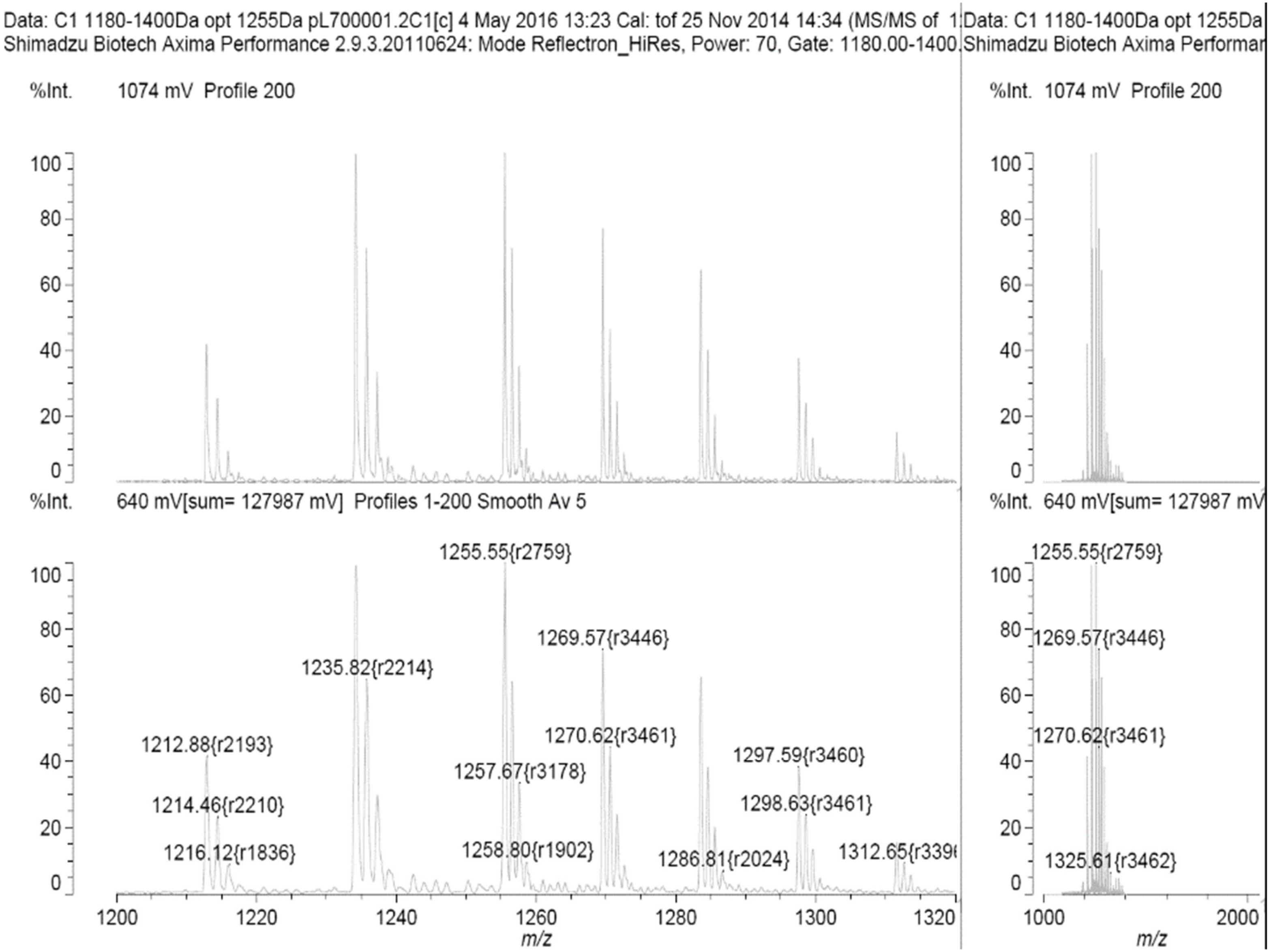

Figure S9. MALDI-TOF spectrum of MβCD

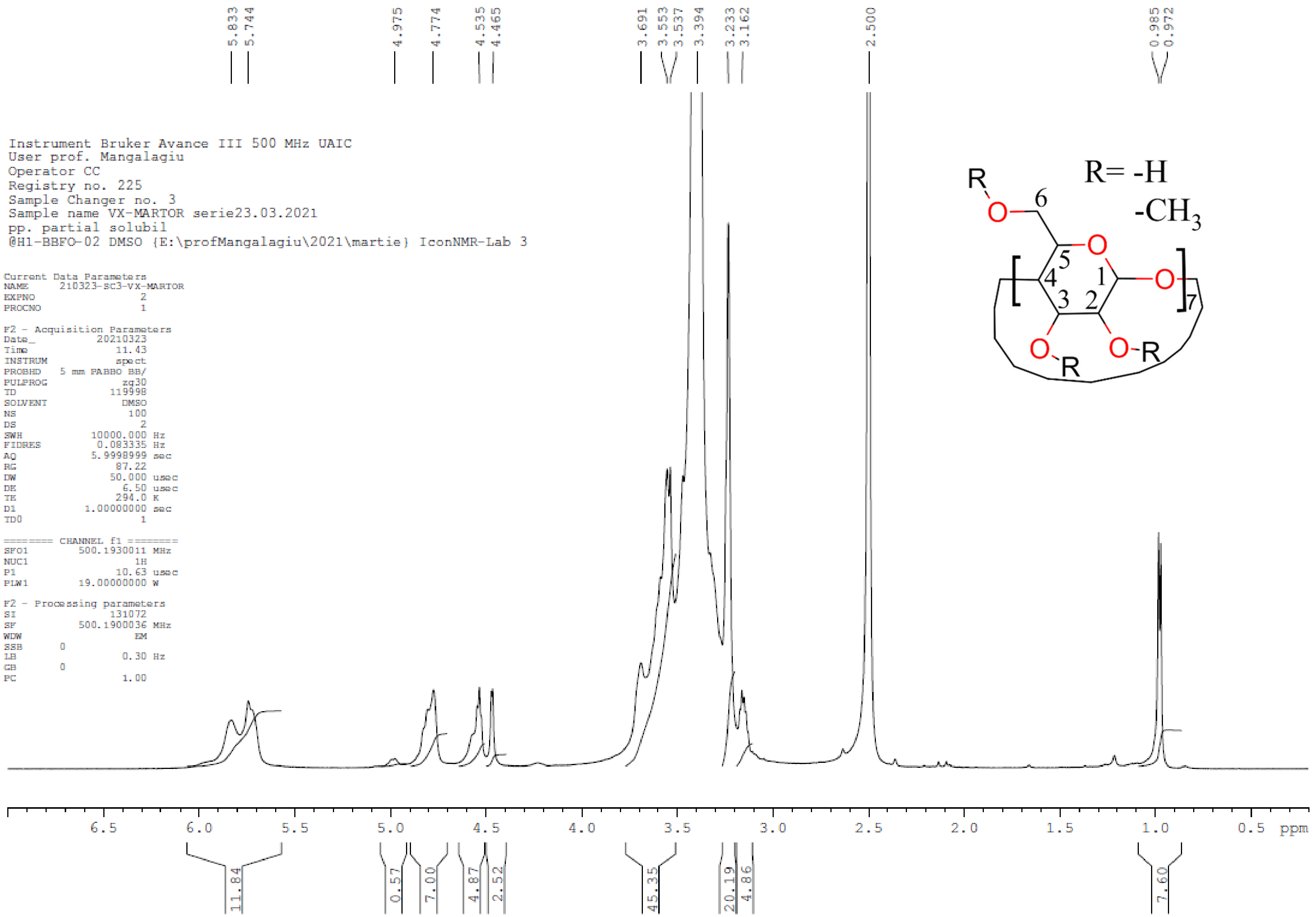

Figure S10. ^{1}H-NMR spectrum of Reference sample

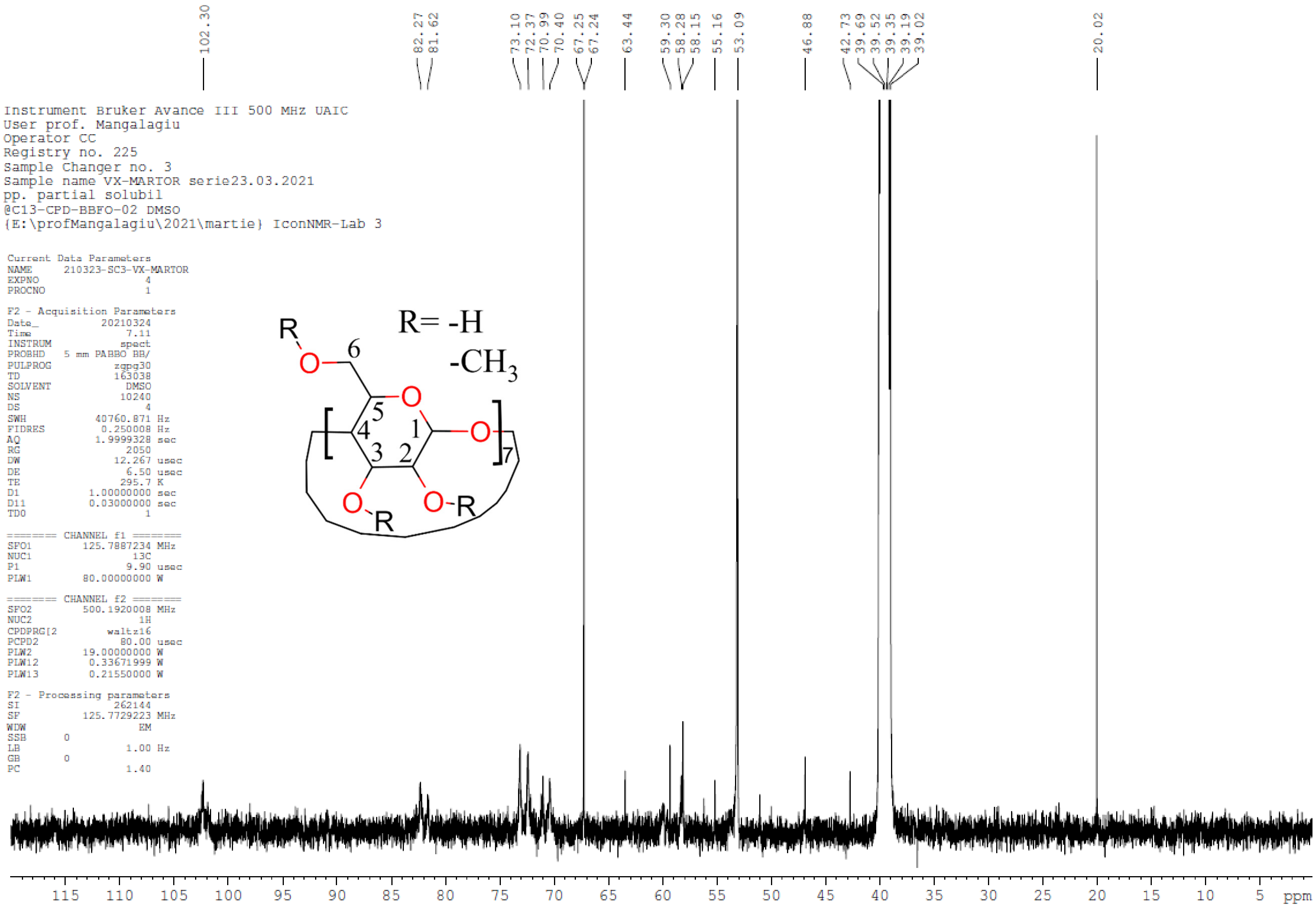

Figure S11. ^{13}C-NMR spectrum of Reference sample

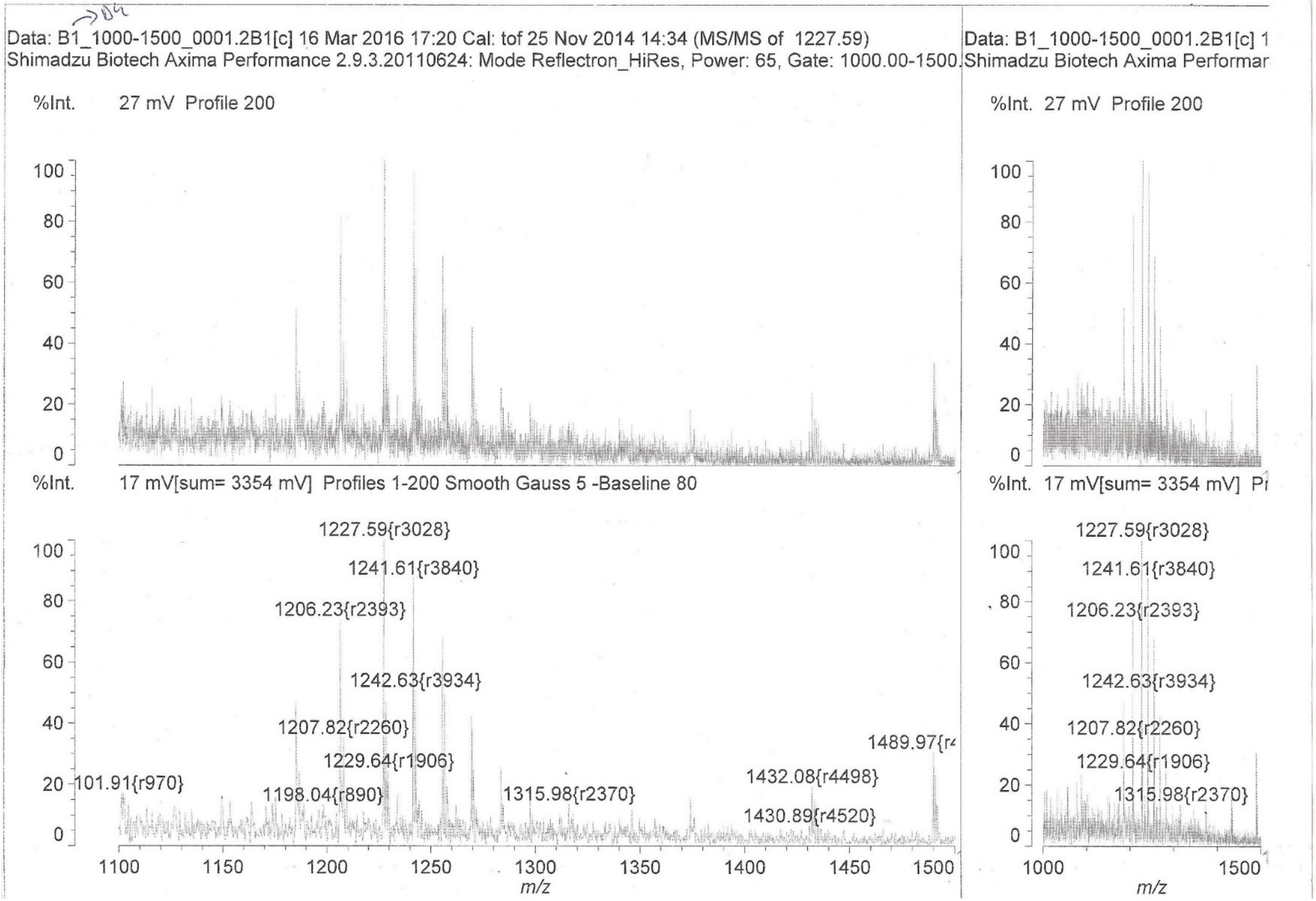

Figure S12. MALDI-TOF spectrum of Reference sample

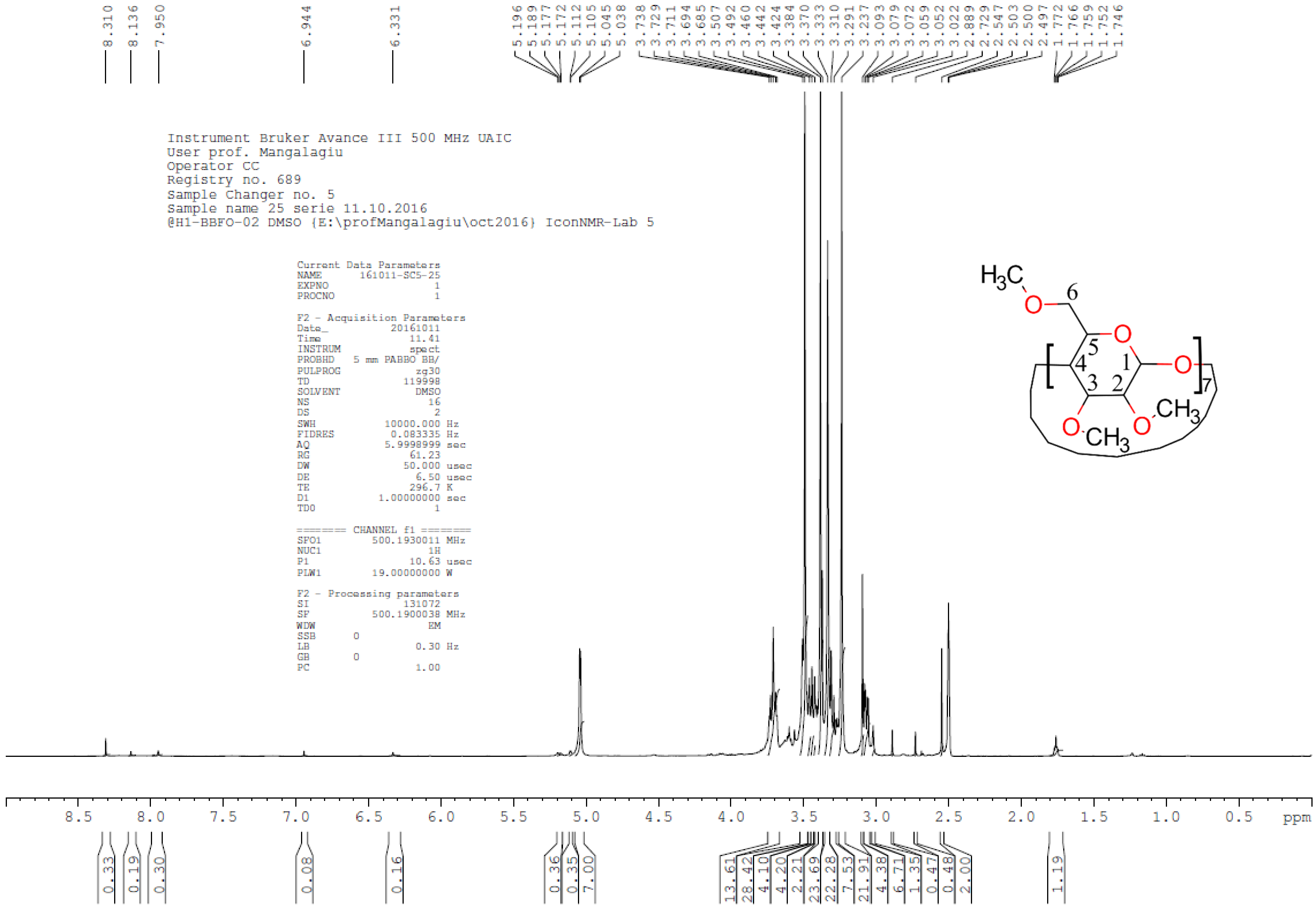

Figure S13. ^{1}H-NMR spectrum of TRIMEB

Table S1. Calculated masses for methylated regioisomers of *β*-CD

CH_3 Groups	Molecular Formula	Monoisotopic Mass	M+H	M+Na	M+K	M+NH4
0 (*β*-CD)	C42H70O35	1134.369764	1135.377589	**1157.359533**	1173.33347	1152.404138
1	C43H72O35	1148.385414	1149.393239	**1171.375183**	1187.34912	1166.419788
2	C44H74O35	1162.401064	1163.408889	**1185.390833**	1201.364771	1180.435438
3	C45H76O35	1176.416714	1177.424539	**1199.406483**	1215.380421	1194.451088
4	C46H78O35	1190.432364	1191.440189	**1213.422133**	1229.396071	1208.466738
5	C47H80O35	1204.448014	1205.455839	**1227.437784**	1243.411721	1222.482388
6	C48H82O35	1218.463664	1219.471489	**1241.453434**	1257.427371	1236.498038
7 (M*β*CD)	C49H84O35	1232.479314	1233.487139	**1255.469084**	1271.443021	1250.513689
8	C50H86O35	1246.494964	1247.502789	**1269.484734**	1285.458671	1264.529339
9	C51H88O35	1260.510615	1261.51844	**1283.500384**	1299.474321	1278.544989
10	C52H90O35	1274.526265	1275.53409	**1297.516034**	1313.489971	1292.560639
11	C53H92O35	1288.541915	1289.54974	**1311.531684**	1327.505621	1306.576289
12	C54H94O35	1302.557565	1303.56539	**1325.547334**	1341.521271	1320.591939
13	C55H96O35	1316.573215	1317.58104	**1339.562984**	1355.536921	1334.607589
14 DIMEB	C56H98O35	1330.588865	1331.59669	**1353.578634**	1369.552571	1348.623239
15	C57H100O35	1344.604515	1345.61234	**1367.594284**	1383.568221	1362.638889
16	C58H102O35	1358.620165	1359.62799	**1381.609934**	1397.583871	1376.654539
17	C59H104O35	1372.635815	1373.64364	**1395.625584**	1411.599522	1390.670189
18	C60H106O35	1386.651465	1387.65929	**1409.641234**	1425.615172	1404.685839
19	C61H108O35	1400.667115	1401.67494	**1423.656884**	1439.630822	1418.701489
20	C62H110O35	1414.682765	1415.69059	**1437.672535**	1453.646472	1432.717139
21 TRIMEB	C63H112O35	1428.698415	1429.70624	**1451.688185**	1467.662122	1446.732789
